# Deconditioning in people living with dementia during the COVID-19 pandemic: qualitative study from the Promoting Activity, Independence and Stability in Early Dementia (PrAISED) process evaluation

**DOI:** 10.1186/s12877-021-02451-z

**Published:** 2021-10-07

**Authors:** Claudio Di Lorito, Tahir Masud, John Gladman, Maureen Godfrey, Marianne Dunlop, Alessandro Bosco, Rowan H. Harwood

**Affiliations:** 1 Division of Rehabilitation, Ageing and Wellbeing, University of Nottingham, Nottingham, NG7 2TU UK; 2 Nottingham University Hospitals NHS Trust, Hucknall Rd, Nottingham, NG5 1PB UK; 3 School of Health Sciences, University of Manchester, Manchester, M13 9PL UK; 4 Division of Health Sciences, University of Nottingham, Nottingham, NG7 2TU UK

**Keywords:** Deconditioning, Physical activity, Exercise, Dementia, COVID-19

## Abstract

**Background:**

Restrictions introduced in response to the COVID-19 pandemic led to increased risk of deconditioning in the general population. No empirical evidence of this effect however has been gathered in people living with dementia. This study aims to identify the causes and effects of COVID-19-related deconditioning in people living with dementia.

**Methods:**

This is a longitudinal phenomenological qualitative study. Participants living with dementia, their caregivers and therapists involved in the Promoting Activity, Independence and Stability in Early Dementia (PrAISED) process evaluation during the COVID-19 pandemic were qualitatively interviewed at two time points: the baseline 2 months after the national lockdown was imposed in England (i.e., May 2020), the follow up 2 months after the first set (i.e. July 2020). The data were analysed through deductive thematic analysis.

**Results:**

Twenty-four participants living with dementia, 19 caregivers and 15 therapists took part in the study. Two themes were identified: Causes of deconditioning in people living with dementia during the COVID-19 pandemic and effects of deconditioning in people living with dementia during the COVID-19 pandemic. A self-reinforcing pattern was common, whereby lockdown made the person apathetic, demotivated, socially disengaged, and frailer. This reduced activity levels, which in turn reinforced the effects of deconditioning over time. Without external supporters, most participants lacked the motivation / cognitive abilities to keep active. Provided the proper infrastructure and support, some participants could use tele-rehabilitation to combat deconditioning.

**Conclusion:**

The added risks and effects of deconditioning on people with dementia require considerable efforts from policy makers and clinicians to ensure that they initiate and maintain physical activity in prolonged periods of social distancing. Delivering rehabilitation in the same way as before the pandemic might not be feasible or sustainable and innovative approaches must be found. Digital support for this population has shown promising results but remains a challenge.

**Trial registration:**

The PrAISED trial and process evaluation have received ethical approval number 18/YH/0059 from the Bradford/Leeds Ethics Committee. The ISRCTN Registration Number for PrAISED is 15320670.

## Background

Sir Muir Gray and Dr. William Bird in an opinion letter published by the British Medical Journal Opinion as the first wave of COVID-19 in the UK was subsiding stated that *“Months of isolation and reduced levels of activity at home will have an immense deconditioning effect on millions of people”* [1].

Deconditioning is a complex process of physiological change after a period of inactivity or sedentary lifestyle [2]. It may result in diminished muscle mass and strength, muscle shortening, alterations of peri-articular and cartilaginous joint structure [2]. The decline in muscle mass and strength is linked to increased risk of falls and hospitalisation, functional decline, frailty, and immobility [2].

A survey carried out in the UK found that 30% of adults aged 50+ reduced activity levels in the first wave of the COVID-19. About 30% of respondents reported that they lacked motivation to exercise [3]. Compared to people without dementia, individuals living with dementia face added challenges to keeping physically active, including impairment of cognitive and motor skills [4–6] and greater apathy or lack of motivation to get active and maintain activity levels [7]. Research has found that during the COVID-19 pandemic more than 50% of people with dementia experienced the worsening or the onset of behavioural disturbances, with agitation/aggression, apathy, and depression being the most common [8, 9].

The risk of deconditioning in people living with dementia was further compounded by governments’ shielding advice for vulnerable people to decrease the risk of contracting the virus. Imposed isolation often resulted in reduced opportunities to do physical activity outdoors and more sedentary behaviour [10]. As reported in the literature, there was also a significant reduction in social support service usage since the outbreak, which greatly affected the opportunities for people with dementia to keep socially engaged and active [11].

The benefits of physical activity and exercise for people living with dementia have long been established. These include the maintenance of physical abilities, independence, and quality of life over time [12]. There is also evidence on the effects of sedentariness, including loss of balance, increased risk of falls and mental ill-health [13]. It has been found that even a few days of sedentary lifestyle generate muscle loss, neuromuscular junction damage and fibre denervation, insulin resistance, decreased aerobic capacity, fat deposition and low-grade systemic inflammation [14]. No empirical evidence on the effect of COVID-19 pandemic on physical activity in a population living with dementia was available at the time of this study.

A unique opportunity to study this phenomenon was presented in the context of the Promoting Activity, Independence and Stability in Early Dementia (PrAISED) randomised controlled trial (RCT) [15]. PrAISED is evaluating an individually tailored physical activity and exercise intervention. Mode of delivery was changed during pandemic lockdown from face-to-face to remote video- or telephone support, where possible. Alongside the RCT, we performed a process evaluation using qualitative interviews to gather data on the impact of the pandemic on physical activity and overall wellbeing [16].

With the aim of improving the ability to reduce the effect of restrictions to activity in people with dementia and how they might be overcome, we used this opportunity to collect and analyse empirical data from the PrAISED process evaluation to answer two research questions:

1. What were the causes of deconditioning for people living with dementia during the COVID-19 pandemic?
2. What were the effects of deconditioning for people living with dementia and their caregivers during the COVID-19 pandemic?

## Methods

This qualitative study made use of data triangulated from interviews with participants living with dementia, their caregivers and the therapists involved in the PrAISED process evaluation during the COVID-19 pandemic. The PrAISED trial and process evaluation have received ethical approval number 18/YH/0059. The ISRCTN Registration Number for PrAISED is 15,320,670.

### Sample

The main researcher (CDL) accessed the PrAISED RCT database - for selection criteria of participants in the RCT see [15]: - and purposively selected participants to ensure an equal representation of geographical location (Nottinghamshire, Derbyshire, Lincolnshire, Somerset and Oxfordshire), gender, residence status (living with caregiver or independently), and RCT arm (i.e., intervention or control group). The caregivers of the selected participants who agreed to take part in the study were also invited to take part in the interviews. No participant living with dementia or caregiver refused to take part. All the therapists involved in the delivery of PrAISED during the COVID-19 pandemic (*n* = 16) were contacted by the main researcher and invited to take part in the study. Only one refused.

### Data collection

Interviews were conducted at two time points: The baseline data collection occurred 2 months after the national lockdown was imposed in England (i.e., May 2020), the follow up 2 months later (i.e., July 2020). The interviews were conducted by a researcher (CDL) remotely, either by phone (i.e., a speaker phone for participants and caregivers to be able to contribute) or video conferencing, depending on the participants’ and therapists’ preference. Prior to the interview session, CDL mailed (or emailed) a copy of the information sheet and consent form. Verbal consent was recorded on tape on the interview day before the interview started from both the participants living with dementia and their caregivers, as per ethical approval number 18/YH/0059 obtained from the Bradford/Leeds Ethics Committee.

The interviews were guided by a topic guide exploring the impact of the lockdown on exercise, activity, and mental well-being (Appendix 1). The topic was developed in collaboration with two Patient and Public Involvement (PPI) contributors with lived experience of caring for someone living with dementia (MG and MD), who helped to make the questions relevant, meaningful, and accessible for the participants. The topic guide was used flexibly, to ensure that new topics emerging during the sessions were explored. The interviews were all digitally audio-recorded on a password protected device approved by governance. Sample size was not defined a priori, and interviews were carried out until conceptual density (i.e., a *sufficient depth* of understanding of the topic under investigation) was reached [17].

### Data analysis

The data were analysed through deductive thematic analysis [18]. Two main themes were established a priori, based on the study objectives: 1. Causes of deconditioning as a result of the COVID-19 pandemic; 2. Consequences of deconditioning as a result of the COVID-19 pandemic. A transcription agency transcribed the interviews verbatim. The transcripts were passed to CDL, who fully anonymised them. CDL then familiarised with the transcripts and noted down preliminary analytical notes.

Coding of a sample of three transcripts was then carried out by CDL and a second researcher within the research team (AB), who extrapolated segments of the transcripts and categorised them into the themes, independent of each other. The two researchers convened to compare their coding and resolve any potential disagreement. CDL then coded the whole set of transcripts in NVivo 12 [19]. Results of the analysis were fed back to the PPI contributors, who provided their comments on the relevance of findings.

## Results

Twenty-four participants living with dementia and 19 caregivers (five participants opted for individual interviews) took part in this study (Table 1). Participants’ mean age was 81 (range: 67–95, SD = 7). Fourteen participants (58%) were male and 19 (80%) lived with the caregiver. Caregivers’ mean age was 73 (range: 50–85; SD = 9). Seventeen caregivers (85%) were spouses of the participant living with dementia and two (15%) children. Twenty-three interviews (96%) were done through speaker phones and one (4%) through webcam.

**Table 1:** Characteristics of participants with dementia and caregivers

Participant ID	Locality	Age	Gender	Living alone?	Type of interview	Caregiver ID	Caregiver’s relationship to participant	Caregiver’s age
P2075	Derby	79	M	No	Dyadic on phone	C2075	Wife	79
P2046		80	M	No	Dyadic on phone	C2046	Wife	84
P2062		87	M	No	Dyadic on phone	C2062	Wife	78
P2069		91	M	No	Dyadic on phone	C2069	Wife	74
P2073		72	F	No	Dyadic on phone	C2073	Husband	75
P1045	Nottingham	95	F	Yes	Dyadic on phone	C1045	Daughter	66
P1051		74	M	No	Dyadic on phone	C1051	Wife	70
P1053		80	F	No	Dyadic on phone	C1053	Husband	85
P1064		79	F	Yes	Individual^a^ on phone			
P1074		84	M	Yes	Individual^a^ on phone			
P4003	Oxford	79	M	No	Dyadic on phone	C4003	Wife	75
P4009		79	M	No	Dyadic on phone	C4009	Wife	71
P4011		89	M	No	Dyadic on phone	C4011	Wife	81
P4019		75	M	No	Dyadic on phone	C4019	Wife	76
P4023		71	M	No	Dyadic on phone	C4023	Wife	58
P3031	Lincoln	67	F	Yes	Individual^a^ on phone			
P3039		72	F	No	Dyadic on webcam	C3039	Husband	63
P3042		83	M	No	Dyadic on phone	C3042	Wife	80
P3044		76	M	No	Dyadic on phone	C3044	Wife	72
P3036		76	F	No^b^	Dyadic on phone	C3036	Daughter	50
P5045	Bath	91	F	No	Dyadic on phone	C5045	Husband	71
P5042		78	M	No	Dyadic on phone	C5042	Wife	84
P5041		89	F	No	Individual^a^ on phone			
P5044		88	F	Yes	Individual^a^ on phone			

^a^ Caregiver was not involved. ^b^ Caregiver moved in during lockdown

Fifteen therapists delivering PrAISED during lockdown were interviewed (Table 2). Four services (80%) delivering PrAISED were represented (i.e., Nottinghamshire, Derbyshire, Lincolnshire, Oxfordshire), but no therapist from Somerset was available for the study. Six therapists (40%) were occupational therapists, five (33%) rehabilitation support workers, and four (27%) physiotherapists. All therapists but one (93%) were female. All interviews were done through the phone.

**Table 2:** Characteristics of therapists and interview sessions

Therapist ID	Site	Profession	Gender	Type of interview
T5	Derbyshire	Occupational Therapist	Female	Phone
T28		Rehabilitation Support Worker	Female	Phone
T8		Rehabilitation Support Worker	Female	Phone
T11	Lincolnshire	Rehabilitation Support Worker	Female	Phone
T13		Rehabilitation Support Worker	Female	Phone
T12		Occupational Therapist	Female	Phone
T14		Occupational Therapist	Female	Phone
T16		Physiotherapist	Female	Phone
T31	Oxfordshire	Physiotherapist	Male	Phone
T30		Occupational Therapist	Female	Phone
T27		Occupational Therapist	Female	Phone
T2	Nottinghamshire	Occupational Therapist	Female	Phone
T3		Physiotherapist	Female	Phone
T4		Rehabilitation Support Worker	Female	Phone
T21		Physiotherapist	Female	Phone

Quotations from participants and therapists are reported in the sections below by identifying their PrAISED number (Tables 1 and 2).

### Theme 1: causes of deconditioning in people living with dementia during the COVID-19 pandemic

Individual-specific socio-demographics and certain environmental, intra and inter-personal variables mediated activity levels. Health issues including physical frailty, stage of dementia, pre-existing psychological morbidity (e.g., depression, anxiety), a history of previous injuries and hospitalisation were all linked to a reduced level of physical activity. This was often compounded by reduced access to health care services during the pandemic:

*“I have had a problem with the left-hand leg and now the right-hand leg, and that’s throwing me out completely. I haven’t been able to get in touch with somebody who could actually help because of lockdown”. P3042, male, 83 years old.*

A lack of internal motivation to exercise was quite common and several participants who lived independently observed that because the therapists were not visiting, they could not rely on the external pressure to exercise. On the contrary, pressure from caregivers helped establish a daily routine of exercise, which was instead linked to prevention of deconditioning:

*I used to do them with an instructor but now I skip the odd ones and I know I shouldn’t be skipping them because it is important. But somebody coming round to knock on the door to take you through them, you just do it” P1074, male, 84 years old.*

*“We (caregiver and participant living with dementia) always have a walk once a day, so it is a routine. Routines are very rigid, and this gives me reassurance that L. is going to do it”. C3044, wife.*

Another key factor affecting exercise levels was the ability of participants to access and use digital support. Traditional means of communication (phone) did not prove effective in supporting participants. Because of age and / or dementia, most participants had difficulties hearing the therapists or communicating. Thus, phone communications ended up occurring only between the therapists and caregivers:

*“Supporting participants through the phone is limited. Over the phone it’s difficult to understand if the participant is hearing or acknowledging what I am saying because you can’t rely on non-verbal cues. So, you end up speaking with the caregiver, which I find totally disempowering for the participant”. T3, PT.*

As a result, the participants who lacked the digital infrastructure or literacy to be able to keep (visual) social contacts with the therapists reported greater apathy and lower motivation to keep active:

*“It is the face-to-face interaction I am missing most. And I haven’t got any enthusiasm to do anything that I would normally do. I am sitting in my garden listening to the birds watching the days go by”. P2073. Female, 72 years old.*

The situation was reversed for participants who felt confident enough to use IT to receive support from the PrAISED therapists through video-consultations. In these circumstances, the social opportunity, support and motivation provided by a professional “outside the family circle” was instrumental for intervention uptake and adherence:

*“Mom just likes to be able to see someone’s face, someone different, she finds it easier to understand the exercises they show her, as opposed to remind her and explain how to do something. Seeing her (the therapist) encourages mom so much!” C3036, daughter.*

The greater need for emotional support among participants was such that the PrAISED therapists reported a paradigm shift in PrAISED delivery, from pre-COVID sessions mostly focused on doing the exercises to a more holistic focus on emotional counselling:

*“It (PrAISED) has actually turned more into a general wellbeing chat, trying to give them positive re-enforcement that they are doing a good job and if they are struggling then encourage them to open up” T3, OT.*

Participants who lived independently (with no in-house informal caregivers) and were in the control group in PrAISED (thus receiving no regular support and contact) were most exposed to the risk of becoming inactive. This suggests that positive encouragement can prevent deconditioning. Another key environmental factor affecting physical activity levels was the availability of a private outdoor area (e.g., garden). Because walking and gardening were the most frequently reported physical activities, having a garden, as opposed to not having a private outdoor space, helped participants keep physically active:

*“We (caregiver and participant living with dementia) have got a fair-sized garden, so myself, I do a lot of gardening which I really enjoy. And I also like sitting and admiring. That really lifts me up” P1053, female, 80 years old.*

One participant reported how staying indoors had greatly limited her chances of doing physical activity, reinforcing the evidence that during the COVID-19 pandemic, not having a private outdoor area might be linked to a greater risk of deconditioning:

*“When this first started out I was walking round our block of flats to keep me supple a bit. But then I got a letter that told me not to go out. I live in blocks of flats and I’m on the downstairs one so I only have a tiny little patio and cannot walk in there” P3031, female, 67 years old.*

In terms of environmental factors, the cancellation of activities in the community drastically reduced the opportunities available for participant to keep active:

*“Lots of place have shut down because of COVID, so all the trips and the outings, I mean we used to do Nordic walking as a group. Now, even if he (participant) wanted to keep doing some things, obviously there is no way” C2046, wife.*

It was observed that deconditioning risk also greatly depended on intra and inter-personal resources to adapt to the changing circumstances. A participant who was carrying out interviews with community dwellers to build a family tree reported he was now doing research online. Another participant, who used to go to the pub for Quiz Friday reported that his family was organising weekly phone quizzes during lockdown. These exciting new projects had prevented a lack of motivation and apathy to keep active:

*“I like to keep things in order, and because I was very busy before (lockdown), I was neglecting the garden. So, I decided right, I am going to concentrate on my garden now, so that is what I have been doing” P1064, female, 79 years old.*

### Theme 2: effects of deconditioning in people living with dementia during the COVID-19 pandemic

Although to various degrees, deconditioning was associated with other negative consequences beyond physical ability including mental wellbeing, affect, motivation to be socially engaged and overall quality of life.:

*“Because I’m in all the time, I don’t do much walking. It makes me very low and so when I go to bed at night I’m thinking about things and everything’s going through my head and instead of one and one making two, one and one make a hundred. My anxiety is out of control” P3031, female, 67 years old.*

It was observed that the impossibility to progress toward physical health goals that required training outside the home (e.g., swimming pools) had made participants apathetic and demotivated to keep active:

*“The initial goals that the participants had set for themselves during lockdown disappeared from the horizon. So, if a participant wanted to start swimming again, during lockdown this was not even a remote possibility. Therefore, it was difficult for them to keep motivated”. T8, RSW.*

Several participants felt that because of a lack or reduction of exercise, they had lost the progress they had previously achieved. Some caregivers agreed that dementia symptoms, such as loss of balance and motor skills had visibly progressed. This was particularly noticeable among the participants who were previously supported face-to-face by the PrAISED therapists:

*“I think the things he was doing (dual task exercises with PrAISED therapist) were keeping his brain and body active. I think he has got a bit slower in his walking and speech now”. C1051, wife.*

This regression made participants lose confidence, resulting in, as eloquently put by one participant, a “lockdown syndrome”, a reluctance and anxiety to leave the home and engage in physical exercise outdoors:

*“The only physical thing that I have been doing lately is walking up and down the garden. I have lost confidence in myself and I need that confidence. I keep saying I should pluck up the courage to go outside of the box that I live in, but I have still not able to get over this locked in syndrome. I tell myself it is what I need to do bit by bit and I am fine with the idea until it comes to actually going”. P2073, female, 72 years old.*

The lockdown syndrome was often compounded by gatekeeping behaviour of caregivers, who were reluctant to encourage the person to go out for fear that they might contract the virus:

*“I still go for a half hour walk around the nearby park everyday, but P. (participant living with dementia) has not been with me. I think he would find it difficult to remember to keep the two-meter rule. I think it will take me a long time to become confident enough to let him out” C2062, wife.*

As a result of some participants becoming more sedentary, caregivers observed a progression of dementia symptoms and an acceleration of the person’s deterioration. The person’s increased dependency needs, and frailty increased the caregivers’ strain and made them anxious about the person’s health:

*“Because mom is dependent on me for shopping, I mostly come every day. This has bought a level of anxiety that wasn’t there before because she’s got quiet frail and I worry I might bring the infection into the house”. C1045, daughter.*

## Discussion

This qualitative study generated empirical evidence on the causes and effects of deconditioning during the COVID-19 pandemic in a sample of participants living with dementia taking part in the PrAISED process evaluation [16]. We found that while the COVID-19 pandemic (and the consequent lockdown) had a negative impact on the quality of life of all participants in the PrAISED RCT, those in the control group (i.e., who did not receive any support from PrAISED therapists) experienced noticeable deconditioning.

Regarding internal validity, this study has certain strengths and limitations. It investigated the phenomenon of deconditioning in real time through a rigorous empirical methodology. Further, it collected data at two time points, to monitor progress of deconditioning over time. It presented a timely contribution to current discourse around the phenomenon of deconditioning by presenting the views of people with lived experience of dementia and their caregivers, promoting representation of their perspectives in research. Further, it introduced the views of service providers too, to add a clinical perspective dimension to the data. Because the study was embedded in an existing trial, the sample might not reflect the wider population of people living with dementia. However, the intention of this study was only to report a real time snapshot of the phenomenon in a specific context, to raise awareness and contribute empirical evidence to the existing discourse on the challenges of people living with dementia during the COVID-19 pandemic.

In relation to external validity, our findings are aligned to the existing literature, suggesting that, although deconditioning has been reported in the general population, having dementia exacerbated the negative effects of deconditioning, particularly in the lack of external support. Apathy, proneness to anxiety and digital illiteracy all contributed to an added risk of becoming inactive [8–11]. However, there were some particular issues unique to dementia. The cognitive demands to maintain social distancing rules was a limiting factor likely to be unique to dementia. The risk that the person with dementia might forget social distancing rules (e.g., forgetting to wear a face mask, to keep social distance, to not shake hands, to wash hands) often made caregivers prone to imposing restrictions. The imposition of restrictions added extra negative effects, as it limited the opportunities for physical activity (outdoors). Further, people with dementia often found it difficult to grasp and remember stay-at-home rules, which led to stress and further compounded demotivation and apathy [20].

In line with the evidence emerging in the literature [21, 22], it was observed that a self-reinforcing vicious cycle was common among the participants, whereby lockdown made the person apathetic, demotivated, socially disengaged, frailer and less confident. This markedly reduced activity levels, which in turn reinforced the effects of deconditioning over time. Without external supporters, most participants living with dementia lacked the motivation / cognitive abilities to keep active. In line with research from the Alzheimer Society of Ireland [22], it also highlighted how minimal but consistent input from therapists drastically reduced the risk and effects of deconditioning. Fully embracing the “use it or lose it” philosophy, most participants in the intervention group acknowledged that “some support is better than no support” and affirmed how helpful having a regular contact with the therapist had been throughout the COVID-19 lockdown.

Looking forward, this study warrants important reflections for clinical practice and policy. The Physiological Society and Centre for Ageing Better presented recommendations from leading scientists and clinicians in the fields of physiology, nutrition, and physiotherapy on how to support older people to remain active and healthy through the pandemic [3]. While the problems identified in this paper have been recognised in the report, the solutions have not been grounded in the experience of people living with dementia. This study illuminates the overlooked but very important issue of people with dementia and provides a grounded evidence-based approach to overcoming the challenges faced by this population.

The added risks and consequences of deconditioning on the population of people living with dementia (compared to the general population) requires considerable efforts to be made from policy makers and clinical practitioners. The National Institute for Health and Care Excellence (NICE) states that *‘there is overwhelming evidence that changing people’s health-related behaviour can have a major impact on some of the largest causes of mortality and morbidity’*. [23] It is therefore a health priority to ensure that people living with dementia initiate and maintain exercise and physical activity in current (or future) circumstances requiring prolonged periods of social distancing.

As advocated in a recently published commentary on rehabilitation needs of older people as a result of the COVID-19 pandemic [24], delivering rehabilitation in the same way as before the pandemic will not be feasible, nor practical or sustainable. Innovative ways and approaches must be found. A recent study looking at the effects of deconditioning reported that even low to medium-intensity resistance exercise, easily implementable at home, have positive effects in preserving neuromuscular, metabolic, and cardiovascular health [14]. The key then, is to promote motivation and engagement to physical activity in the absence of home-visits from trained professionals.

Tele-rehabilitation has shown promising results. During the COVID-19 pandemic, virtual support has been used to reduce behavioural problems [25], and to deliver cognitive therapy [26]. Television-based assistive integrated technology has been used to provide health and social support [27]. However, there has been limited implementation of tele rehabilitation as a strategy against deconditioning. Our study adds to the mounting evidence [ 22] that maintaining consistent face-to-face (remote) contact between professionals and people living with dementia is an effective strategy to keeping them motivated and active, compared to phone support (or no support at all).

While other studies have advocated for digital remote support to keep people with dementia active and engaged throughout the pandemic, [20, 28, 29] we believe that much remains to be done in this area. We found that people living with dementia still experience challenges to accessing digital tele-rehabilitation, given, among other factors, a lack of digital infrastructure, information technology literacy and cognitive impairment. Similar findings have been echoed in a recent Dutch study highlighting barriers to the uptake of communication technologies in nursing homes during the COVID-19 pandemic [30].

In order to make tele-rehabilitation really equitable for all clients living with dementia, partnership with caregivers is crucial, as they facilitate the process of remote support by, for example, operating IT devices, facilitating communication, reporting accurate information, and supervising the person while they exercise (thus minimising risk and injuries). Because caregivers may experience added care burden in the context of stay-at-home regulations, [31] consideration is to be made about their need to receive specific practical and psychological support to buffer the effect of public health restrictions and reduced access to paid care [11, 32]. This would potentially limit caregiver burden and make caregivers more willing and able supporters of the persons they care for. A recent study on caregiver burden during COVID-19 reported that caregivers advocated the use of video-consultations and telephone-based support to have their needs met in these challenging times [33].

## Conclusion

New interdisciplinary prevention approaches against deconditioning are urgently needed that account for the drastic changes that social distancing situations are likely to generate [34]. In line with similar attempts to introduce digital technologies in supporting people living with dementia during the COVID-19 pandemic, [35, 36] this study showed that provided the proper infrastructure (e.g. digital hardware and software, live support for users to navigate the system, partnership with caregivers) and balanced integration with the (still preferred) face-to-face interaction, [37] in times of need, people living with dementia are willing to use technology [ 38] to prevent the risks and effects of deconditioning. Investment in digital connectivity, as well as in providing support to enable this population to use tele-rehabilitation must therefore be scaled up, to ensure readiness for future challenges.

## Data Availability

The datasets generated and analysed during the current study are not publicly available to safeguard anonymity of participants, but are available from the corresponding author on reasonable request.

## Abbreviations

PrAISED: Promoting activity, independence and stability in early dementia
RCT: Randomised controlled trial
PPI: Patient and public involvement
SD: Standard deviation
NICE: The national institute for health and care excellence
